# Isoniazid Killing of *Mycobacterium smegmatis NADH Pyrophosphatase* Mutant at Single-Cell Level using Microfluidics and Time-Lapse Microscopy

**DOI:** 10.1038/s41598-017-11503-7

**Published:** 2017-09-07

**Authors:** Meltem Elitas

**Affiliations:** 1 School of Life Sciences, École polytechnique fédérale de Lausanne (EFPL), 1015 Lausanne, Switzerland; 2 Present Address: Sabanci University, Orta Mah. No: 27, Tuzla, Istanbul, 34956 Turkey

## Abstract

We introduce single-cell analysis for isoniazid-treated *Mycobacterium smegmatis* mutant, *msm1946-NADH pyrophosphatase*, using microfluidics and automated time-lapse microscopy. Mycobacterial NADH pyrophosphatase isoforms play an important role for the mechanism of isoniazid and ethionamide activation. Our single-cell analysis revealed important insights on isoniazid killing mechanism that was masked by traditional killing assays, raised significant questions related to viable but non-culturable subpopulation of cells, and existing methods that defines minimum inhibitory concentration of drugs. The major goal of this study was quantitatively analyze bacterial cell parameters to obtain high-resolution data for the time evolution of antibiotic killing at the single-cell level. The presented tools and methods could be applied to the closely related organisms to provide more detailed information for the design and employment of antibiotic treatments.

## Introduction

Investigation of eukaryotic cells using micro-and nanotechnologies in conjunction with automated time-lapse microscopy encouraged the application of similar technologies for microbial cells. Cutting-edge micro-and nanofabrication processes allowed manufacturing of tremendous microenvironments for the microorganisms and advances in high-resolution imaging techniques endorsed observation of their fascinating world. Finally, a framework has been established for a new era of modern microbiology research^1–4^.

During this revolution, first investigations were achieved via real-time observation of prokaryotic single-cells on thin agar pads^5^. Afterwards, these setups were improved to provide changing environmental conditions for long-term, live-cell imaging^6^. They accomplished to inspire invention of microfabricated single cell chemostats^7^ and bioreactors^8^. These single-cell tools have incorporated with different imaging techniques^2, 3, 9, 10^ to follow single-molecules inside a single bacterium^11^. Thanks to this technical modernization, unrevealed mysteries of microbial world started to be visible and questionable. Cellular heterogeneity and stochasticity of bacterial populations have been investigated^12, 13^, new bacterial species have been discovered^14^, special subpopulations of microbiota and their culturing conditions have been identified and noteworthy contributions have been made to biodiversity^15^. Wu and Dekker^16^ presented an excellent review for further knowledge of the use of nanofabricated structures and microfluidic devices for bacterial studies.

These stimulating research and developments have encouraged scientists to apply microfabricated tools for tackling mycobacterial enquiries such as obtaining quantitative^3^, single-cell resolution data for mycobacterial genetics^17^, heterogeneity^18–21^, its interaction with host-cells^17^, antibiotic-tolerance^18, 20, 21^ and antibiotic-resistance^22^ mechanisms. Among these studies, Balaban’s and McKinney’s findings have challenged the current antibiotic-killing mechanisms, particularly for one of the first-line tuberculosis drug, isoniazid (isonicotinic acid hydrazide, INH) and its impact on slowly growing mycobacterium subpopulations^18, 19, 23^.

INH has been one of the most effective and widely used of all antitubercular drugs since 1952. It is a pro-drug that is oxidatively activated *in vivo* by the katG-encoded mycobacterial catalase-peroxidase to generate covalently modified INH-NAD adduct^24^. INH-NAD adduct inhibits InhA^25^, an enoyl reductase that is a member of the type II dissociated fatty acid biosynthesis pathway. INH interferes with the biosynthesis of mycolic acids, the very long chain fatty acid components of the mycobacterial cell wall^26–29^. In addition to these findings, still several characterization studies for the mechanism of action of INH has been performed and the obtained knowledge is still being used to identify new drug compounds^30^. One of the pioneering studies presented mycobacterial NADH pyrophosphatase isoforms that plays an important role in a novel mechanism for INH and ethionamide (second-line therapy reagent, ETH) inactivation^31^.

In this study, we will present a single-cell analysis method for isoniazid-treated *Mycobacterium smegmatis* mutant, *msm1946-NADH pyrophosphatase*, using microfluidics and automated time-lapse microscopy. Our quantitative single-cell analysis results will continue to challenge current dogma that explains how INH kills mycobacteria.

## Results

### *M. smegmatis msm1946*::*Tn* mutant

The *M. smegmatis msm1946*::*Tn* mutant was studied using a genetic approach in conjunction with microfluidics-based time-lapse fluorescence microscopy. The most important phenotype of this mutant was the increased killing rate during exposure to INH or ETH. The *msm1946*::*Tn* transposon mutant grew indistinguishably from wild-type bacteria in standard 7H9 liquid medium (Supplementary Figure 1). The ability of the mutant cells to form colonies on standard LB solid medium was also indistinguishable from wild-type cells. Therefore, the increased killing response of this mutant was not due to any general growth defect or hypersensitivity to other stresses that were tested (Supplementary Figure 5). Moreover, complementation and overexpression studies of the *M. smegmatis msm1946*::*Tn* mutant was also confirmed its special phenotype (Supplementary Figures 2–4).

### Batch culture killing and drug specificity

**Figure 1:**
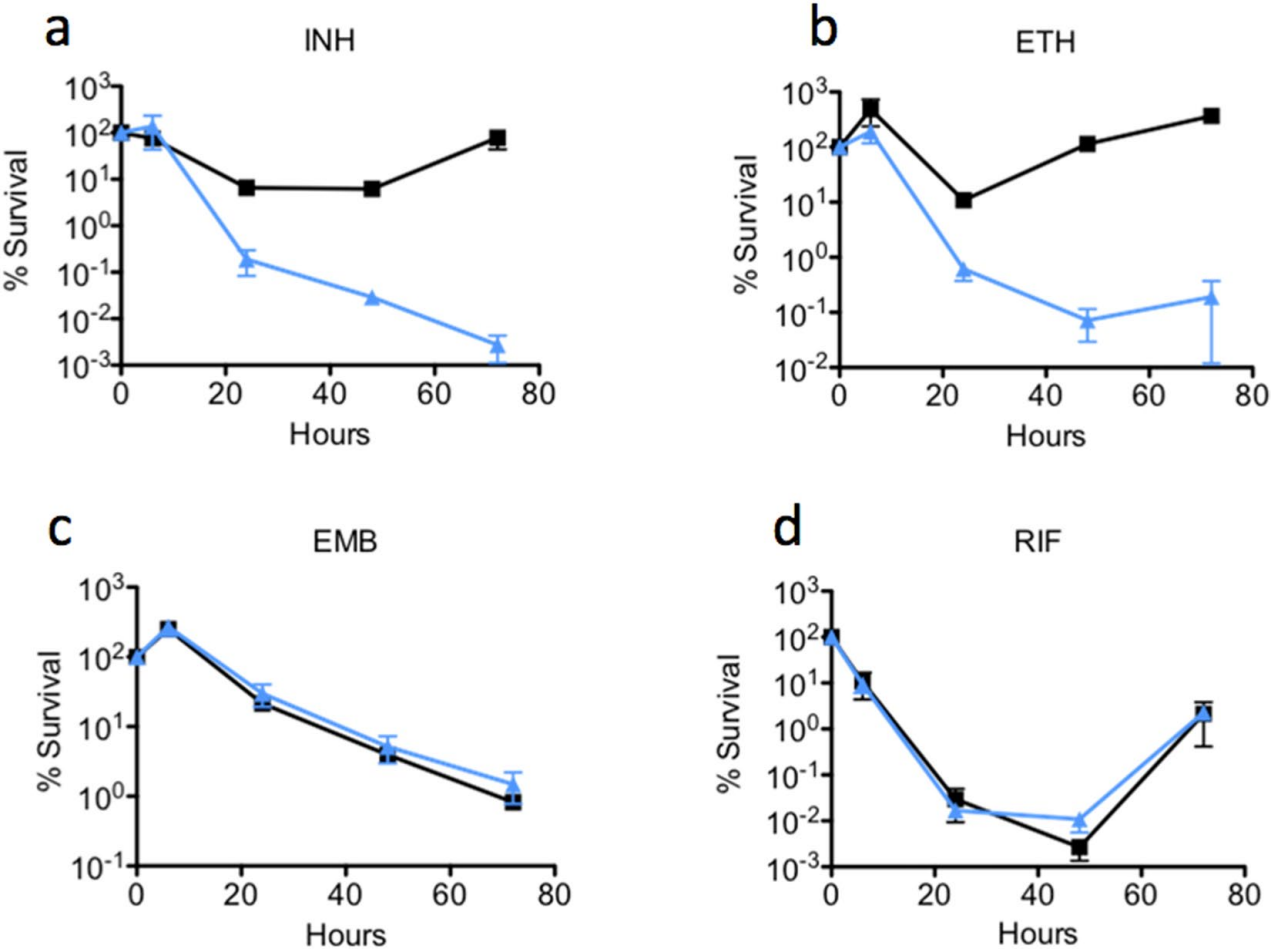
Drug specificity and killing assay for the *msm1946*::*Tn* transposon mutant. *M. smegmatis* wild-type (black line with squares) and *msm1946*::*Tn* transposon mutant (blue line with triangles) strains were exposed to (**a**) INH 50 µg/ml, (**b**) ETH 200 µg/ml, (**c**) EMB 5 µg/ml, or (**d**) RIF 200 µg/ml. Serial dilutions of the drug-treated cultures were used to determine the percent survival (CFU assay). Results are means ± standard errors from three independent cultures.

Antibiotic specificity of the *M. smegmatis msm1946*::*Tn* was studied using anti-tubercular compounds; INH, rifampicin (RIF), ethambutol (EMB), and ETH (Fig. 1).

The impact of msm1946 disruption on drug-mediated killing was found to be specific to INH and ETH. Both INH and ETH are pro-drugs that must be activated by covalent linkage to NAD (INH-NAD and ETH-NAD adducts). In contrast, the *msm1946*::*Tn* mutant and wild-type cells were killed with similar kinetics when incubated with EMB or RIF (Fig. 1).

When INH- and ETH-mediated killing of the *msm1946*::*Tn* mutant were quantified, there was a significant killing difference between the *msm1946*::*Tn* mutant and wild-type cells. However, the *msm1946*::*Tn* cells neither became resistant after 72 hours of drug treatment nor grew back when INH was removed from the medium, Table 1.

**Table 1:** Specificity of drug-mutation interaction. Comparison of persistence phenotype rates, defined as the fractional survival ratio (FSR).

	FSR ± SE WT	FSR ± SE *msm1946*::*Tn*
**INH**	1 ± 0.16	0.004 ± 0.008***
**EMB**	1 ± 0.63	1.3 ± 2.16
**RIF**	1 ± 0.001	4.06 ± 0.005
**ETH**	1 ± 3	0.0006 ± 0.04**

The FSR of the *msm1946*::*Tn* transposon mutant divided by the FSR of the wild-type control at 48 hours of exposure to: INH 50 µg/ml, ETH 200 µg/ml, EMB 5 µg/ml, or RIF 200 µg/ml. *p* values were calculated using the Student**’**s unpaired *t*-test (***p < 0.0001, **p < 0.01, *p < 0.05).

### Monitoring the INH-mediated killing using microfluidics in conjunction with time-lapse microscopy

To verify the results of batch culture assays at the single-cell level analysis, a series of time-lapse INH exposure experiments were performed using the single-cell microfluidic platform^27^.

The microfluidic device consisted of a Polydimethylsiloxane (PDMS) microfluidic network to feed the cells. The cells were seeded between a coverslip and semipermeable membrane, which prevents movement of cells with fluid flow and provides two-dimensional monolayer growth. Repetitive, single-cell resolution images of the cells were obtained without any background problems those exist in many microfluidic-microscopy setups. Furthermore, the device was very user-friendly for operation^27^. In each experiment, multiple x-y points initially containing single cells were programmed, images of the cells in these points were recorded every 15 minutes using an Olympus IX75 motorized inverted microscope equipped with a Hamamatsu ORCA-AG CCD camera and a 100 × oil-immersion objective (UPLFLN). The images were recorded on the phase and fluorescence channels (TRIS-red: 150 µs, and GFP-green: 150 µs). The microfluidic chip was mounted on the motorized stage of the microscope, which was inside a temperature control chamber.

**Figure 2:**
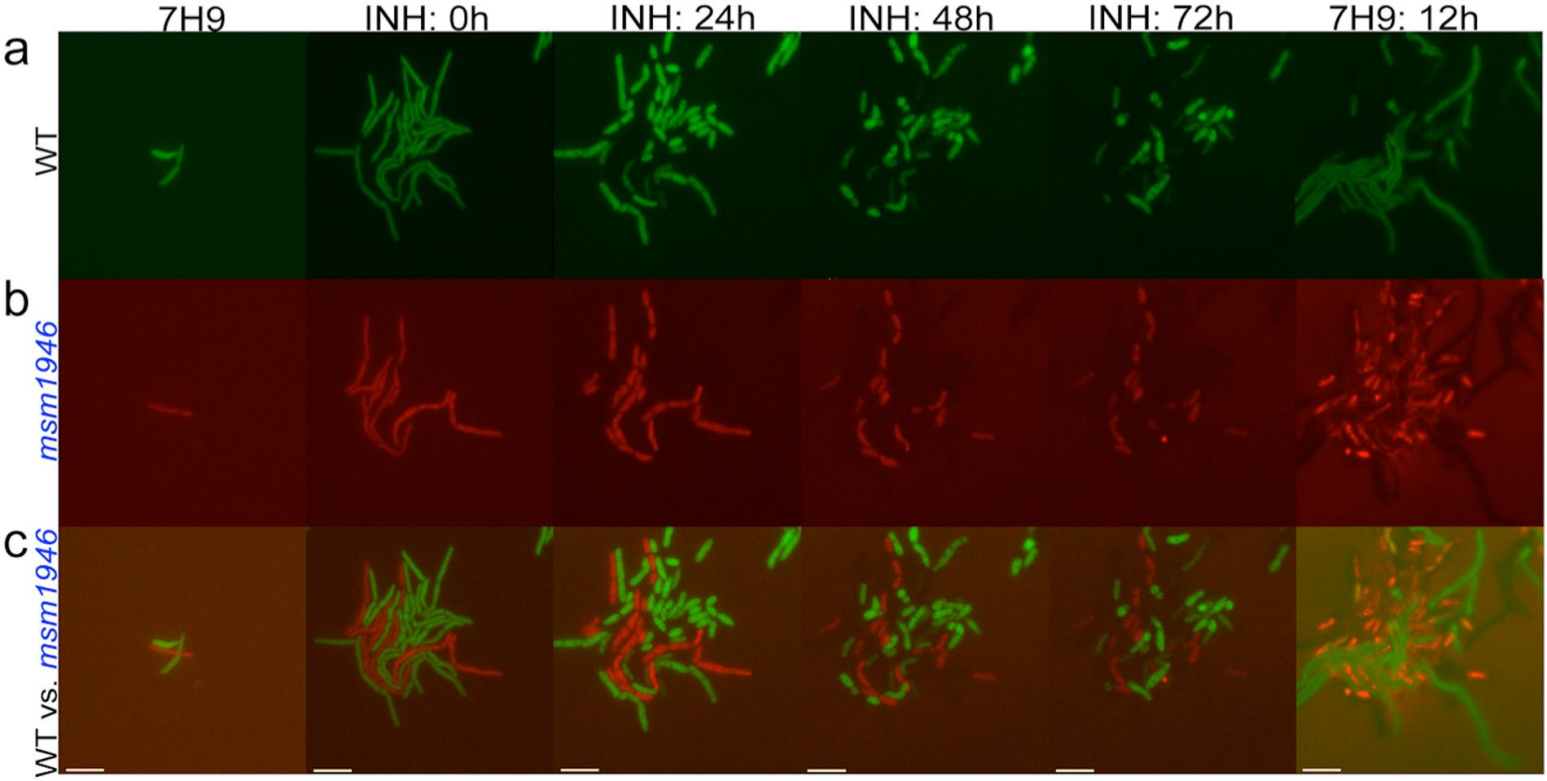
Behavior of wild-type vs. *msm1946*::*Tn* mutant using microfluidics and single-cell time-lapse microscopy. (**a**) WT (green), (**b**) *msm1946*::*Tn* (red), (**c**) merged WT (green) vs. *msm1946*::*Tn* (red). The experiment includes growth (7H9), drug killing (INH:0 h – INH: 72 h, INH-50 µg/ml) and washing (7H9: 12 h) steps.

For the microfluidic-microscopy experiment, constitutively green fluorescent protein (GFP) expressing, single wild-type cells (WT) and constitutively red fluorescent protein (RFP) expressing, single *msm1946*::*Tn* mutant cells were mixed and simultaneously introduced into the same microfluidic device. Our aim was to exclude the possibility that differences in the growth environment were the cause of the persistence defect seen in previous assays. The microfluidic-microscopy experiment consists of three steps. First, the growth of the cells was observed in standard 7H9 medium (Fig. 2, 7H9-INH: 0 h). Second, the cells were exposed to INH for three days to monitor their differential killing (Fig. 2, INH: 0 h–72 h). Third, INH was withdrawn and the cells were fed with normal 7H9 medium for 12 hours to observe recovery from the antibiotic exposure (Fig. 2, INH: 72 h-7H9: 12 h). Finally, an end-point staining was performed with propidium iodide (PI) to label INH-damaged cells (Fig. 2, 7H9: 12 h–bright red cells).

During the first part of the experiment, both WT cells and the *msm1946*::*Tn* mutants grew in regular 7H9 medium. When the drug exposure period started both wild-type cells and the *msm1946*::*Tn* mutants were killed. The *msm1946*::*Tn* mutant cells underwent cytolysis to a much lesser extent than WT cells. Subpopulation of the *msm1946*::*Tn* mutant cells remained physically intact and brightly fluorescent throughout the period of the drug exposure. When the drug was removed from the flow medium, wild-type cells recovered and resumed growth and division, whereas the *msm1946*::*Tn* mutant cells failed to resume growth. These results were consistent with the results of the batch culture experiments.

### Single-cell analysis

**Figure 3:**
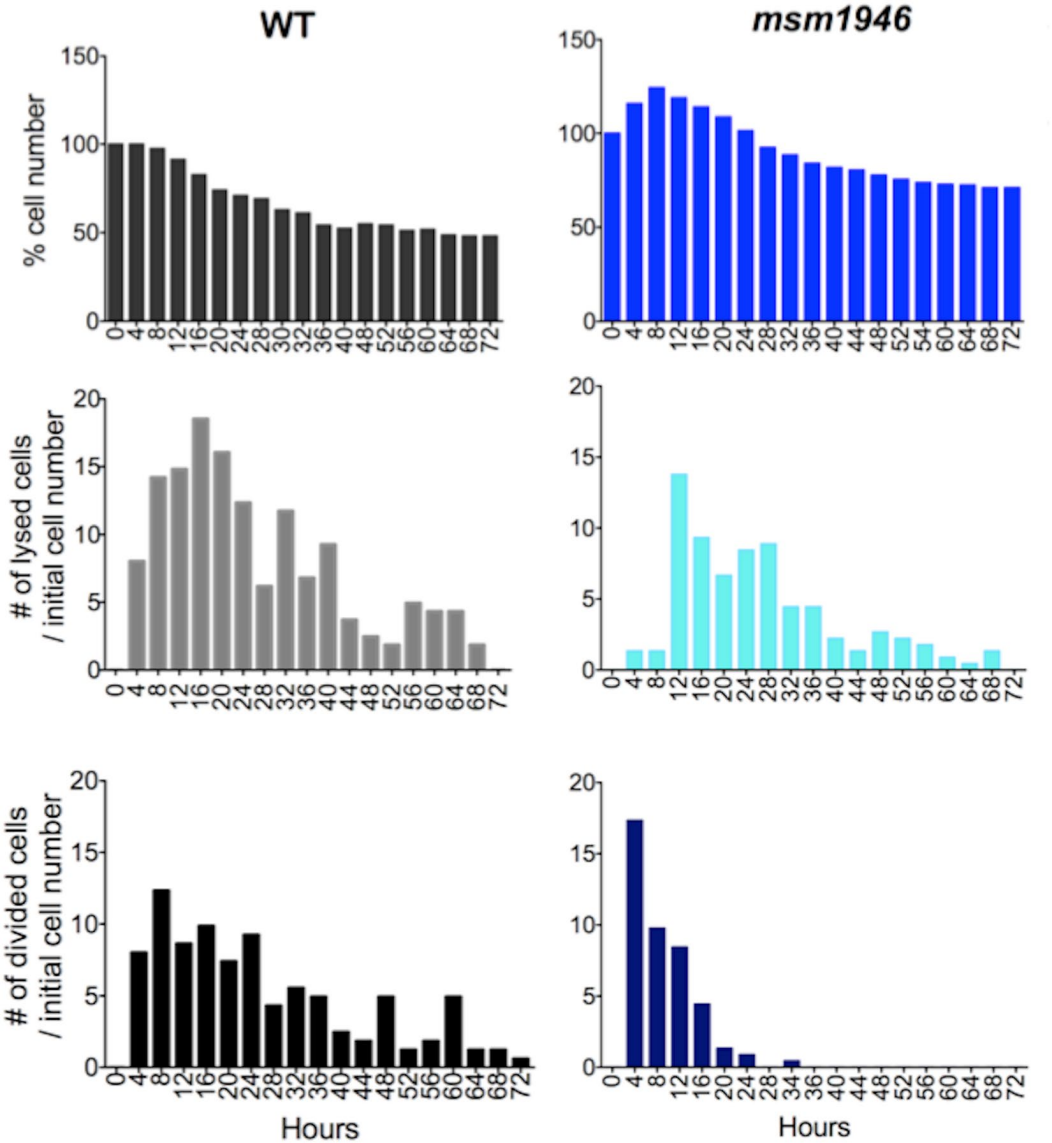
Quantitative single cell analysis based on time-lapse fluorescent movies of WT *M. smegmatis* vs. the *msm1946*::*Tn* mutant during the 72 hours of INH 50 µg/ml exposure in the single cell microfluidic device. First row demonstrates the cumulative behavior of the cells, the second row demonstrates number of normalized lysis, and the last row illustrates number of normalized division events.

In order to obtain quantitative data from the time-lapse experiments, single-cell resolution image analysis was performed using ImageJ. The cytolysis and division events were separately counted for WT and the *msm1946*::*Tn* mutant cells using both phase and fluorescence image stacks for each time frame. Then, all the events were gathered for four hours time period. Figure 3 shows the cumulative cell number change, the number of normalized lysis and the number of normalized division differences between WT and *msm1946*::*Tn* mutant cells during the 72 hours of INH exposure.

The percent (%) cell number graph of wild-type cells presented a clear biphasic killing profile during the drug exposure. The number of cells increased during the first 8 hours of drug exposure due to continued cell division in the presence of the drug. This initial “lag phase” was followed by a rapid “killing phase” in which many cells underwent cytolysis at rates that were much higher than the underlying rate of cell division. The “killing phase” was followed by a prolonged “persistence phase” in which cell numbers were relatively stable due to roughly balanced rates of cell division and cell lysis (Fig. 3).

The percentage (%) cell number graph of msm1946::Tn mutant cells also showed an increase in the cell numbers during the first 8 hours of drug exposure. Afterwards, rapid killing ensured due to an increased number of lysis events and decreased number of division events. By 32 hours of drug exposure, division events ceased while cell lysis events continued at a low rate.

At the end of the drug exposure period, the number of physically intact *msm1946*::*Tn* mutant cells was higher than the number of intact wild-type cells; this was largely because the *msm1946*::*Tn* mutant cells initially exhibited a low level of lysis compared to WT cells (Fig. 3).

### Phenotypic subpopulations of INH-exposed cells

**Figure 4:**
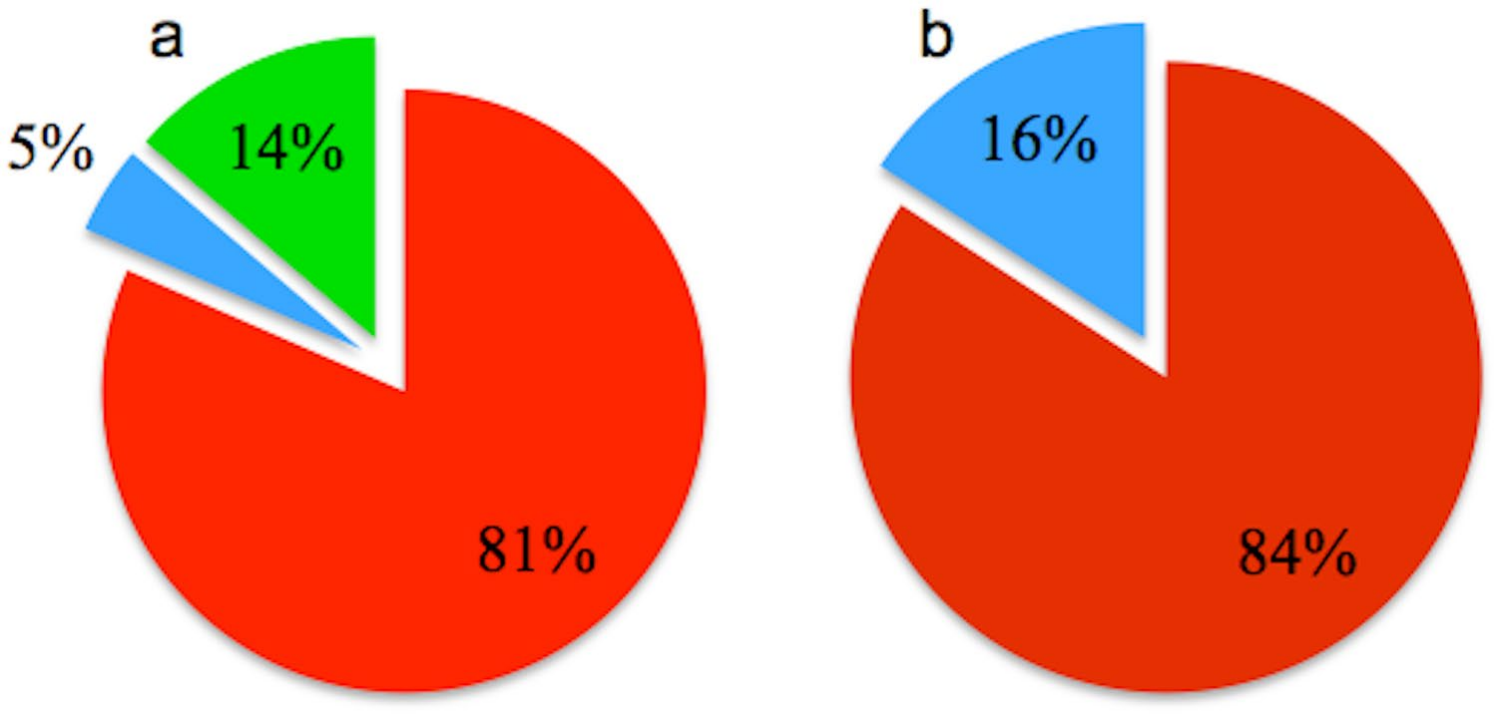
Phenotypic subpopulations of INH-exposed cells. (**a**) 81% of the wild-type cells were PI-positive and non-growing (red); 5% of the wild-type cells were PI-negative and non-growing (blue); 14% of the wild-type cells were PI-negative and growing after INH washout (green). (**b**) The *msm1946*::*Tn* mutant cells exhibited 84% PI-positive and nongrowing (red); 16% of PI-negative and non-growing (blue) phenotypes.

In order to clarify whether the intact non-growing cells were dead or damaged by INH, an end-point staining was performed using PI, which selectively stains cells with a damaged cell wall permeability barrier. In order to perform this assay, upon removal of the drug, cells were incubated in 7H9 medium containing PI for 10 hours, then the PI was removed from the medium and the cells were fed with standard 7H9 medium for an additional 10 hours. The time-lapse images stacks were generated to quantify the number of PI-positive cells, the number of PI-negative cells, and the number of cells that recovered growth. The number of the cells in each subpopulation was manually counted from the time-lapse image stacks using ImageJ. Figure 4 presents these subpopulations.

This assay reported the number of intact wild-type cells upon INH exposure as 512; 417 of them were PI-positive, 24 of them were PI-negative, one of them lysed, and 70 of them resumed growth after INH washout.

Regarding the *msm1946*::*Tn* mutant: the number of intact cells following INH exposure was 2,312; 365 of them were PI-negative, 1,947 of them were PI-positive, and none of the cells were seen to lyse or regrow after INH washout.

The subpopulation of intact and PI-negative cells were observed for both wild-type (5%) and the *msm1946*::*Tn* mutant cells (16%). Apparently, this subpopulation of cells was non-culturable since these cells did not resume growth in the microfluidic device upon drug removal. Additionally, the *msm1946*::*Tn* mutant cells also did not form colonies on agar plates in traditional batch culture killing assays (CFU). Although the intact/PI-negative cells did not resume growth after drug washout, they would ordinarily be scored as “viable” based on maintenance of fluorescent protein expression and failure to stain with PI.

## Discussion

In this study, the *msm1946*::*Tn* mutant was studied using a genetic approach in conjunction with microfluidics-based time-lapse fluorescence microscopy. The most important phenotype of this mutant was the increased killing rate during exposure to INH or ETH without any general growth defect (The complementation and overexpression studies were presented in the supplementary document).

The live-cell imaging experiments provided the real-time kinetic information of INH killing at single-cell resolution. The results of these studies raised fundamental and compelling questions about the dynamics of bacterial persistence and the biphasic killing profile of INH. The kinetics of mono-drug killing observed in batch culture experiments was broadly consistent when the lumped behavior of single cells was considered. However, the quantitative single-cell analysis clarified the underlying persistence-phase killing kinetics as consisting of balanced cell division and cell lysis. Moreover, the single-cell analysis paradoxically demonstrated that the mutant cells, although defective for persistence and regrowth after drug washout, nonetheless displayed lower rates of cytolysis (scored as loss of fluorescence) during the antibiotic exposure as compared to wild-type cells (Figs 3 and 4, Supplementary movie).

As mentioned above, although INH killed the *msm1946*::*Tn* mutant cells more than wild-type cells, the mutant cells showed lower rates of cytolysis. These results are in conflict with the currently accepted model of INH killing, i.e., the “unbalanced growth” model. This model postulates that under normal growth conditions, cell wall biosynthesis and cell growth are balanced, whereas, in the presence of INH, a progressively defective cell wall, due to inhibition of mycolic acid biosynthesis, is incapable of balancing the pressure of cell growth and fostering cells lysis^26^. This model was established based on batch culture experimental results. However, it has been recently challenged by time-lapse observations. The observed rates of lysis of the *msm1946*::*Tn* mutant was lower than the rates of lysis of wild-type cells (Figs 3 and 4, Supplementary movie). These investigations suggest that this model is not adequate enough to account for the mechanism of INH-mediated killing. Despite the widespread use of INH, how INH kills bacilli still remains obscure. INH enters the mycobacterial cell through passive diffusion^32^ and is thought to kill only dividing bacteria; no killing is observed when the cells are not actively dividing either in stationary phase or in anaerobic conditions^33^. The action of INH is multi-phasic: it is bacteriostatic for the first 24 hours, then the action becomes bactericidal during the initial killing phase of anti-tuberculosis therapy, and its activity is dramatically reduced thereafter during the persistence phase^18^. This reduction in activity is not associated with the emergence of isoniazid-resistant mutant strains but, rather, with the selection of phenotypically tolerant persisters^34, 35^.

The msm1946 gene is annotated as encoding NADH pyrophosphate (NudC), which catalyzes the hydrolysis of NAD(H) to AMP (Adenosine Mono Phosphate) and NMN (Nicodinamide Mono Nucleotide)^31^. Thus, it was hypothesized that the *msm1946*::*Tn* mutant cells might accumulate abnormally high levels of NADH. INH and ETH are pro-drugs that require activation by formation of covalent INH-NAD or ETH-NAD adducts; in the case of INH, this reaction is catalyzed by bacterial catalase-peroxidase (KatG)^31^. Elevated levels of NADH in the *msm1946*::*Tn* mutant could then contribute to enhanced formation of the INH-NAD adduct (active form of the drug), resulting in enhanced killing the *msm1946*::*Tn* mutant. Wang and co-workers carried out a comparative analysis of mycobacterial NADH pyrophosphatase isoforms ^31^. Their results are consistent with ours and helped to explain why the M. tuberculosis rv3199c deletion strain did not show the enhanced killing response to INH, i.e., because in most strains of M. tuberculosis the enzyme is inactive due to a single-nucleotide polymorphism (P237Q). When we combine the results from our studies and those of Wang *et al*., a mechanistic explanation for the phenotype of the *msm1946*::*Tn* transposon mutant emerges. This mutant presumably does not produce active NADH pyrophosphatase (NudC) due to the transposon insertion in the msm1946 gene. Therefore, NADH pyrophosphatase would not be available to degrade the activated forms of INH-NAD or ETH-NAD. Thus, as we hypothesized, increased levels of the INH-NAD or ETH-NAD adduct in the mutant causes increased killing by INH or ETH. Consistent with this interpretation, overexpression of the msm1946 gene in wild-type *M. smegmatis* resulted in the opposite phenotype, i.e., reduced killing by INH or ETH, presumably due to enhanced degradation of the INH-NAD and ETH-NAD adducts (Supplementary document).

It is likely that the M. tuberculosis rv3199c deletion strain did not show the increased killing kinetics during INH exposure because the M. tuberculosis NudC has a polymorphism at the highly conserved residue 237^27^. The P237Q polymorphism prevents dimer formation, resulting in a loss of enzymatic activity. Because the enzyme in M. tuberculosis would not play a role in degradation of the INH-NAD or ETH-NAD adducts even in wild-type cells, complete loss of this enzyme by deletion of the rv3199c gene would not be expected to alter the response to INH or ETH.

The deletion and overexpression of NudC in M. bovis BCG background, which expresses the P237 form of the enzyme, provided results consistent with the *M. smegmatis* data. Specifically, deletion of the nudC gene increased killing by INH or ETH, whereas overexpression of nudC had the opposite effect^31^.

Although the mechanistic basis of the *msm1946*::*Tn* mutant’s phenotype seems clear, there remain some interesting questions about the phenotype of this mutant. For example, although the *msm1946*::*Tn* mutant cells were unable resume growth after INH washout, cell lysis was actually reduced in the mutant compared to the wild-type control. Thus, our studies demonstrate that the mutant generates forms that resemble “viable but non-culturable” cells (i.e., cells that are physically intact, PI-negative, GFP-bright, and non-growing) at an elevated rate as compared to wild type. In future it will be important to determine whether these cells are truly “dead” or whether they retain metabolic activity and, potentially, the ability to resume growth if provided with the correct stimulation.

## Conclusions

The aim of this work was to develop modern tools and methods that would provide quantitative data on the different aspects of the phenomenon of antibiotic-killing mechanisms. The movies and quantitative data obtained in this work provide new insights into the dynamic behavior of how INH kills cells and why a fraction of cells evades killing. The currently accepted unbalanced growth model of INH-mediated killing postulates cytolysis as the proximal mechanism of cell death. In contrast, although INH apparently killed the mutant more than wild-type that was confirmed at the population level in batch culture assays, at the single-cell level the mutant cells actually lysed at a lower rate than wild-type cells.

Paradoxically, when PI staining was used to assess the integrity of the cell wall, a greater number of intact and PI-negative cells was detected in the *msm1946*::*Tn* mutant cells as compared to the wild-type control. This subpopulation of intact and PI-negative cells might correspond to so-called “viable but nonculturable” phenotypic variants, which could account for the decreased rates of regrowth after INH washout.

The presented results are examples of how microtechnology can contribute to the discipline of microbiology to yield a deeper understanding of a specific phenotype. The obtained data are an interrogation more than a conclusion; therefore, there are many future avenues of this investigation. The presented methodology and outcomes could also be applied to single-cell analysis of other biomedically relevant pathogens, including Escherichia coli, Staphylococcus aureus, Pseudomonas aeruginosa, Candida albicans, M. tuberculosis. Achieving a better understanding of the mechanistic basis of persistence and drug-killing action could help to identify strategies that influence the susceptibility of persistent infections to drug treatments.

## Materials and Methods

### Media and chemical reagent

Middlebrook 7H9 medium is prepared using g Middlebrook 7H9 powder (Difco) supplemented with 10% Middlebrook albumin-dextrose-saline (Difco), 0.5% glycerol and 0.1% Tween-80 (Sigma-Aldrich). Luria-Bertani (LB) medium was made with Luria Miller broth (Sigma-Aldrich). Agar plates were prepared with 15 g/l Bacto agar (Sigma-Aldrich) and 15.5 g/l Luria Miller broth base in water. Propidium iodide (PI) was stored frozen as a 2 mM stock solution. PI staining solution was made at 0.4 µM in 1X PBS 0.02% Tyloxapol and stored at 4 °C.

### Culture/Growth conditions

A spectrophotometer (Thermo Scientific biomate 5) was used to determine turbidity of cultures by measuring optical density (absorbance at 600 nm), using 1 ml cuvettes. Dilutions were made in 7H9, to keep the reading in the linear range of the spectrophotometer.

### Drug susceptibility assays

#### Colony forming unit (CFU)

Serial 10-fold dilutions were made using 100 µl culture and 900 µl 1X PBS- 0.025% Tween solution in 5 ml polypropylene tubes. 100 µl of appropriate dilutions were plated onto agar-based media to insure that the serial dilutions would give at least one countable plate (giving 30–300 countable colonies). Then, plates were incubated to enumerate colonies formed. ***Kill Curves***. Mycobacterial cells were grown to mid-log phase (OD_600_ of 0.5–1) and diluted to OD_600_ of 0.05, corresponding to ~10^7^ CFU/ml, in fresh 7H9 medium. Then different drugs were added into each sample. Unless otherwise specified, antibiotics were used at the following concentrations: INH: 50 µg/ml, RIF: 200 µg/ml, ETH: 200 µg/ml, BTZ: 100 ng/ml, EMB: 5 µg/ml, Ciprofloxacin: 1.5, 3 µg/ml, Streptomycin: 50 µg/ml. For combinatorial therapy; INH-ETH: INH-50 µg/ml and ETH- 200 µg/ml, INH-RIF: INH-50 µg/ml and RIF- 200 µg/ml. Then CFU assays were performed and plates were incubated at 37 °C. Colonies were counted at day 3 and 4.

#### Minimum inhibitory concentration (MIC)

Agar proportion method was used. Mycobacterial cells were grown to mid-log phase (OD_600_ of 0.5–1) and diluted to OD_600_ of 0.05 in fresh 7H9 medium. Then 10-fold serial dilutions were plated on LB agar solid plates containing various concentrations of INH (2-fold serial dilutions: 100–0.024). Plates were incubated at 37 °C. MIC was defined as the lowest concentration of drug required the see minimum bacterial CFU.

### Genomic DNA preparation

Genomic DNA was extracted from 10–20 ml cultures at OD600 ~0.5–1.0 using lysozyme treatment, proteinase K digestion, Cetrimide (hexadecyltrimethylammonium bromide (CTAB)) saline, and chloroform extraction^36^.

### RNA isolation and gene expression

*In vitro* grown bacteria were spun down for 15 min at 4000 rpm at 4 °C in a Sorvall tabletop centrifuge. Bacterial pellets were resuspended in 500 µl Trizol reagent and transferred to 2 ml O-ring screw cap tubes containing ~250 μl zirconia beads. Trizol immersed samples were lysed with the beads in a BioSpec Bead-Beater 2 times for 1 min with a 2 min interval on ice. The samples were spun for 30 sec at 12,000 g at 4 °C to pellet down the beads. Then, the supernatants were transferred to clean microfuge tubes. The second extraction started with addition of 50 µl of 1-bromo-3-chloropropane into the supernatants and vortexing for 15 sec at room temperature. Next, the sample was incubated for 10 min at room temperature and then spun at 12,000 g for 15 min at 4 °C. The upper phase of the supernatants were recovered and extracted with isopropanol following the same conditions and washing steps previously. Finally, the supernatant was discarded and washed with 70% ethanol. The pellet was air-dried, and resuspended in 50 µl of DEPC water (DNA-free) and stored overnight at 4 °C. Residual DNA was removed by treatment with DNA-free or TurboDNA-free (Invitrogen) as specified by the manufacturer.

### Transposon (Tn) Mutagenesis and construction of transposon mutant library

The fMycoMarT7 transposon donor phagemid, which carries a selectable marker for kanamycin resistance, was provided by Dr. Eric Rubin (Harvard School of Public Health). The phagemid was used to mutagenize a strain of *M. smegmatis* mc^2^155 expressing DsRed from a strong constitutive promoter, carried on a single-copy plasmid integrated at the chromosomal attB site; the plasmid also carries a hygromycin resistance marker. Cells were grown at 37 °C to late-log phase (OD_600_ 1.0) in 7H9 medium, washed twice with MP buffer (50 mM Tris, pH 7.5, 150 mM NaCl, 10 mM MgSO_4_, 2 mM CaCl_2_), and resuspended in 1/10^th^ original volume in MP buffer. 10^10^ PFU (plaque-forming units) of phage per ml of original culture were added for 3 hours at 37 °C. Infected cells were plated on LB agar plus 50 µg/ml hygromycin and 25 µg/ml kanamycin. After 3 days, well-isolated colonies were individually picked with sterile toothpicks into 96-well microtiter plates containing 7H9 plus 50 µg/ml hygromycin and 25 µg/ml kanamycin. Plates were incubated at 37 °C for 5 days. Original 96-well master plates were stored at −80 °C in 15% glycerol while the copy plates were inoculated, grown in 7H9 media, and then used for spotting.

### Identification of transposon mutants

*M. smegmatis* genomic DNA was isolated and digested with BamHI. The digested DNA was ligated with T4 ligase (NEB) overnight and transformed into Pir1 competent *E. coli* cells (Invitrogen). Kanamycin-resistant colonies were selected, and plasmid DNA was isolated and sequenced using the primer cttctgagcgggactctgggg, which hybridizes near one end of the fMycoMarT7 transposon.

### Statistical analysis

Student’s unpaired t-test (two-tailed) was used to assess statistical significance of pairwise comparisons. P values < 0.05 were considered significant. P values were calculated using GraphPad QuickCalcs online software (http://www.graphpad.com/quickcalcs/ttest1.cfm?Format=SD).

### Live-cell imaging

In each experiment, multiple xy points initially containing single cells growing within the device were programmed and images were recorded every 15 minutes using an Olympus IX75 motorized inverted microscope equipped with a Hamamatsu ORCA-AG CCD camera and a 100 × oil-immersion objective (UPLFLN), inside a temperature controlled chamber held at 37 °C. Images were recorded on the fluorescence (TRIS-red: 150 µs, and GFP-green: 150 µs) and phase channels.

### Image analysis

Time-lapse fluorescent movies were used for image analysis. Number of lysis and division events was counted for every 4-hour time frame. ImageJ cell counter was used and figures plotted in Prism4.

### Microfluidic platform

The microfluidic platform^27^ consisted of a PMDS chip patterned with a microfluidic network (micro channels: 50 µm × 50 µm), a semipermeable membrane (Spectra/Por Dialysis membrane, Spectrum Lab, MWCO: 8), a circular coverslip (#1), a PMMA (Poly(methyl methacrylate)) holder, and inlet and outlet tubing (Standard Silicone Tubing, ID: 0.03 in (0.076 cm), OD: 0.065 in (0.165 cm), HelixMark). Cells are seeded between the coverslip and the semipermeable membrane, which prevents movement of cells with fluid flow and enables repeated imaging of single cells growing in a two-dimensional monolayer using a high magnification (100 × objective) inverted microscope. Operation of the device and medium switching were very simple and practical. The medium flow was controlled by a syringe pump (World Precision Instruments) with a flow rate of 25 µl/min.

## Electronic supplementary material

Supplementary document
