# Supplementary material for: Isoniazid Killing of Mycobacterium smegmatis NADH Pyrophosphatase Mutant at Single-Cell Level using Microfluidics and Time-Lapse Microscopy
Source: Sci Rep. 2017 Sep 7;7:10770. doi: 10.1038/s41598-017-11503-7 (PMC5589797; doi:10.1038/s41598-017-11503-7)
Supplement: Supplementary file 1 — Supplementary document [file 41598_2017_11503_MOESM1_ESM.pdf]

Isoniazid Killing of *Mycobacterium smegmatis NADH Pyrophosphatase* Mutant at Single-Cell Level using Microfluidics and Time-Lapse Microscopy

Meltem Elitas[1,2]

Author's current address: [1]Sabanci University, Orta Mah. No: 27, Tuzla, Istanbul, 34956, Turkey. melitas@sabanciuniv.edu.

Author performed the work: [2]School of Life Sciences, École polytechnique fédérale de Lausanne (EFPL), 1015 Lausanne, Switzerland.

Supplementary
Growth of the msm1946::Tn transposon mutant. The *msm1946::Tn* transposon mutant grew indistinguishably from wild-type bacteria in standard 7H9 liquid medium (Supplementary Figure 1).

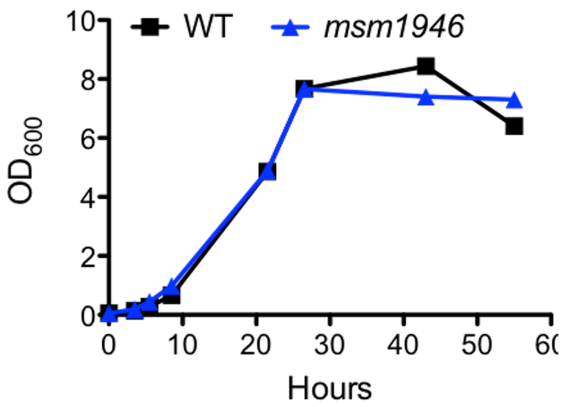

Supplementary Figure 1 Growth of the *msm1946::Tn* transposon mutant in 7H9 medium. Growth was monitored by measuring culture turbidity (OD_{600nm}) at the indicated time points. Results are representative of at least two experiments.

Complementation of the *msm1946::Tn* Mutant. For complementation of the *msm1946::Tn* transposon mutant, the msm1946 gene was PCR-amplified from wild-type M. smegmatis genomic DNA (Supplementary Figure 2). The full-length gene was cloned into integrating plasmid as described below.

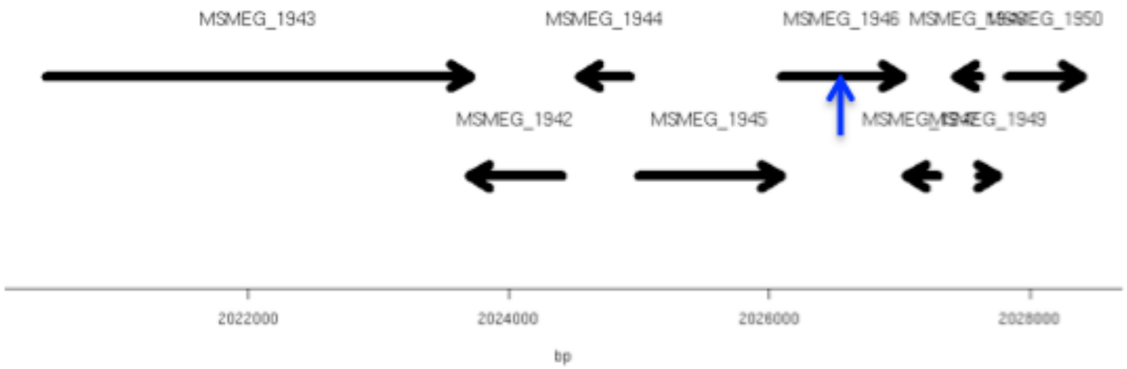

Supplementary Figure 2 Chromosomal locus encoding the *msm1946* gene in *M. smegmatis.* Location of the transposon insertion indicated by vertical arrow.

Integrative complementation (*pND200_Strep_msm1946*). Complementation of the *msm1946::Tn* mutant with a single-copy attB-integrating plasmid (pND200_Strep) containing the intact msm1946 gene was performed. The INH-mediated killing response of the *pND200_Strep_msm1946* strain followed wild-type kinetics only for the first 24 hours of drug

exposure; thereafter, the killing kinetics were slower than wild type (Supplementary Figure 3). The minimum inhibitory concentrations for INH of the integrating complemented and over-expression strains were higher than 100 µg/ml (compared to 5 µg/ml for wild-type bacteria).

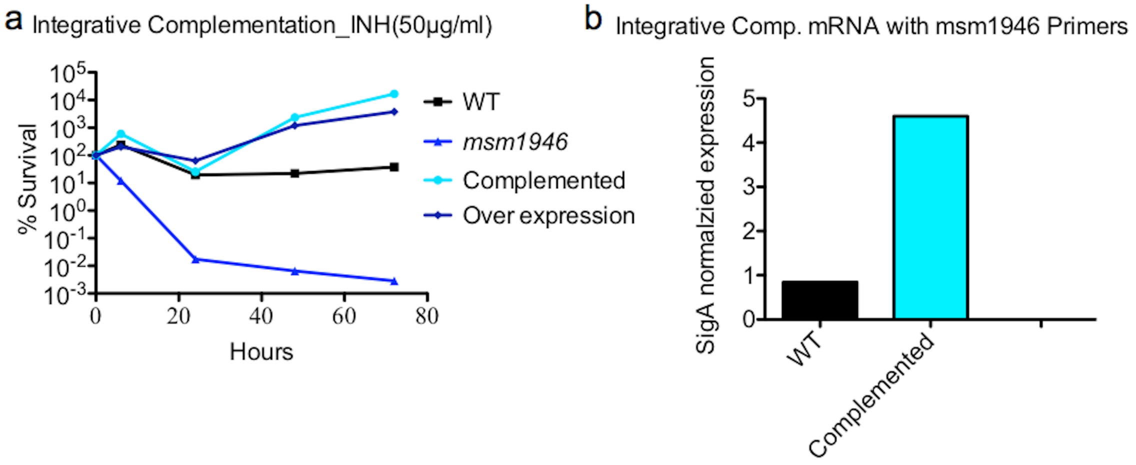

Supplementary Figure 3 Integrative complementation of *msm1946* mutant and its over expression in WT *M. Smegmatis.* **a)** INH mediated killing for WT *M. smegmatis* (black), persister down mutant (triangle), its complementation with an integrative plasmid (circle) and over expression of the gene *msm1946* in WT background (diamond). **b)** Real-time RT-qPCR of the *M.smegmatis* wild-type strain (black), and its integrative complementation (light blue). Cells were grown until the exponential growth phase was reached (OD_{600}, approximately 0.5 - 0.8). RNA was isolated and transcription of *msm1946* gene was monitored by real-time RT-qPCR using specific primer set for *msm1946*. For each gene, expression levels normalized to constitutive gene, *SigA*.

Nonetheless, the integrative complemented or overexpression strains exhibited imapired growth in 7H9 medium. Failure to achieve appropriate levels of complementation might still be attributable to the unbalanced expression of the msm1946 gene from the strong *hsp60* promoter of the pND200 integrating vector.

mRNA levels of inhA and katG in the msm1946::Tn mutant. Since KatG is involved in the activation of INH and *inhA* is the target gene for INH, the relative mRNA levels of *katG* and *inhA* were measured for the wild-type, *msm1946::Tn* mutant, and integratively-complemented *msm1946::Tn* strains. *katG* and *inhA* mRNA levels were not altered in the *msm1946::Tn* mutant compared to wild-type. However, both of these genes were expressed at slightly reduced levels in the complemented strain (Supplementary Figure 4). As a control, *msm1946* levels were also measured (Supplementary Figure 4).

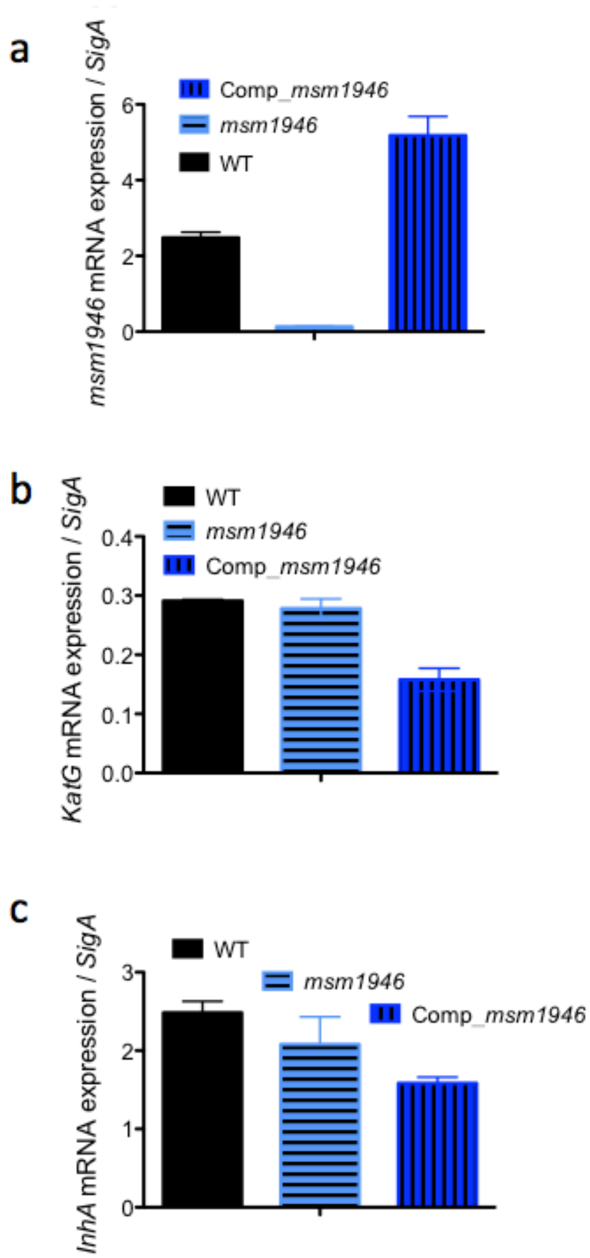

Supplementary Figure 4 *katG* and *inhA* mRNA expression levels in wild-type, *msm1946::Tn* transposon mutant, and *msm1946::Tn* complemented strains. Bars represent ratio of *msm1946* **a)**, *katG* **b)**, and *inhA* **c)** mRNA levels relative to *sigA* mRNA (control). *sigA* is a constitutively transcribed gene encoding the housekeeping sigma factor of RNA polymerase. The number of transcript copies was determined by qRT-PCR using gene-specific primers listed.

INH-Resistence Test. In the context of INH killing, *the msm1946::Tn* transposon mutant was never observed to acquire INH resistance after 48 hours of drug treatment, unlike wild-type cells, which typically "bounced back" after 48 hours due to emergence of drug-resistant variants. In order to determine whether the *msm1946::Tn* mutant was capable of becoming INH resistant, the

mutant and wild-type cells were grown to OD_{600nm} 0.4 and 10-fold serial dilutions were plated onto LB solid medium containing INH at 0, 0.6, 6.0, or 60 µg/ml). The plates were incubated at 37°C degrees for several days to permit colonies to form. Single colonies of mutant and wild-type cells were picked from the plates containing 0.6 µg/ml INH, which exhibited colony formation for both the mutant and wild-type cells (Supplementary Figure 5). Next, single colonies were inoculated in standard 7H9 medium and stocks of the cultures were prepared and named WT_0.6 and *msm1946_0.6*. Growth of these strains in 7H9 medium (no drug) was indistinguishable from wild-type (Supplementary Figure 5).

Thus, neither the WT_0.6 nor the *msm1946_0.6* strain was resistant to INH at 50 µg/ml although these strains readily grew on LB plates containing INH at 0.6 µg/ml. To confirm that these strains were not strongly INH resistant, the MIC values were determined using the micro-dilution method (see the Materials and Methods). Although the MIC value of the parental *msm1946::Tn* mutant was 0.39 µg/ml INH, the *msm1946_0.6* strain managed to grow in 7H9 medium containing INH at 12.5 µg/ml, suggesting that this strain might have acquired low-level resistance to INH.

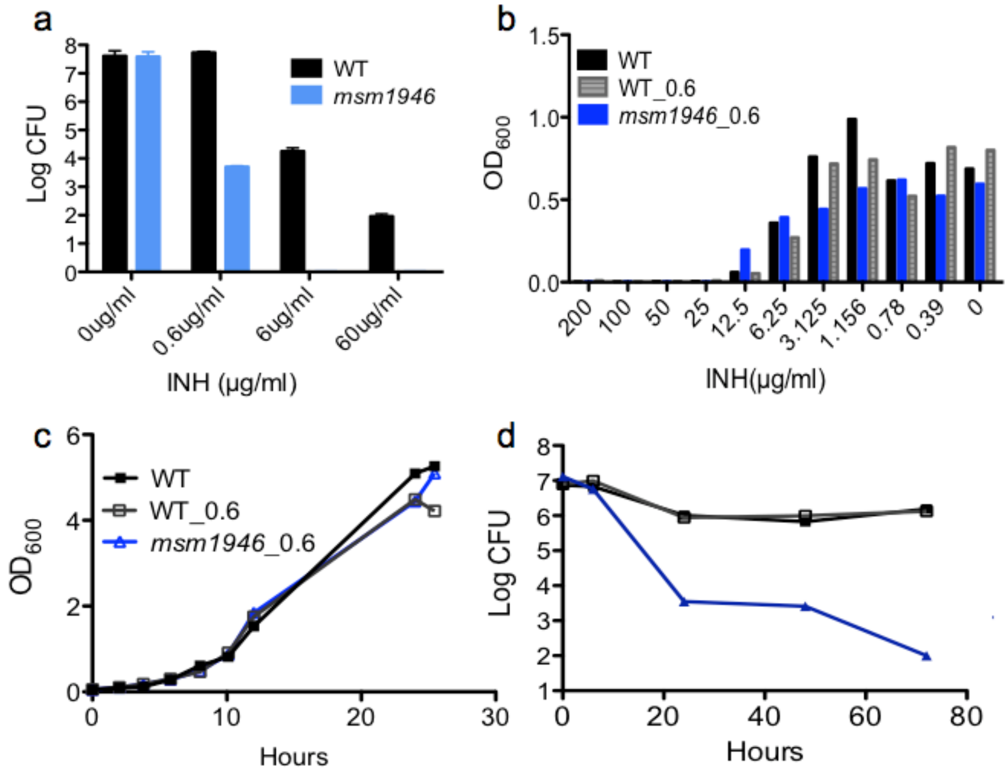

Supplementary Figure 5 a) The frequencies of INH resistance were determined by plating wild-type and *msm1946::Tn* mutant cells onto LB solid medium containing INH at 0, 0.6, 6.0, or 60 µg/ml. **b)** The *WT_0.6* and *msm1946_0.6* single colonies were picked from LB agar plates containing INH 0.6 µg/ml. *WT_0.6* and *msm1946_0.6* cells grew like wild-type in standard 7H9 medium (no drug). Growth was

monitored by measuring culture turbidity (OD_{600nm}) at the indicated time points. **c)** Drug specificity and INH-mediated killing for the *WT_0.6* and *msm1946_0.6* strains. *M. smegmatis* wild-type and the *WT_0.6* and *msm1946_0.6* strains were exposed to INH 50 µg/ml. Serial dilutions of the INH-treated cultures were plated at the indicated time points to determine the surviving CFU count. Results are the means ± standard errors of three independent cultures. d) The INH MICs of the *msm1946_0.6* and *WT_0.6* strains were not different than wild-type. The MIC values were determined by measuring growth by monitoring turbidity (OD_{600nm}) of the cultures grown in 7H9 medium containing the indicated concentrations of INH.

Stress Response. In addition to drug specificity, sensitivity of the *msm1946::Tn* mutant to other stresses was assessed, including thermal stress exposure to the detergent sodium dodecyl sulfate (SDS) acidic pH, and nutrient starvation by incubation of cells in phosphate-buffered saline (PBS) (Supplementary Figure 6). The *msm1946::Tn* transposon mutant did not show increased sensitivity to any of these stresses (i.e., the mutant was equally sensitive as wild-type cells), which suggests that hypersensitivity to the mutant had INH sensitivity otherwise it behaves like WT, which encourages to investigate the underlying INH killing mechanism for this mutant.

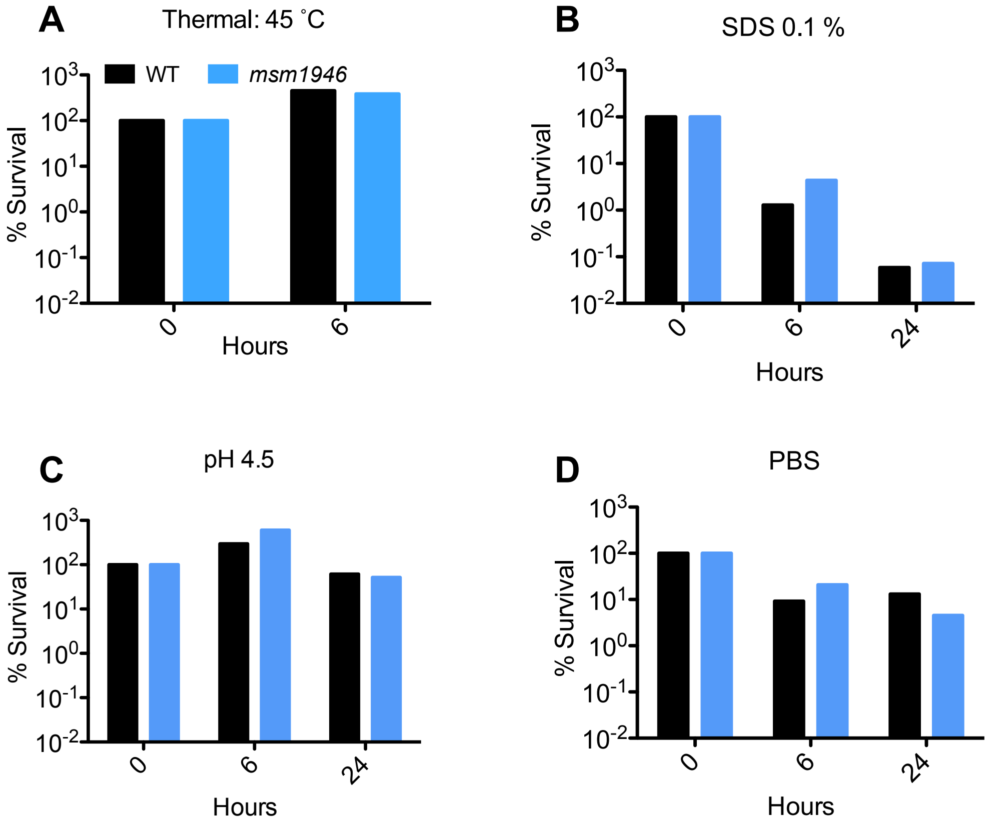

Supplementary Figure 6 Sensitivity of *the msm1946::Tn* mutant and wild-type cells to thermal, SDS, pH, and PBS. Percent survival of WT and the msm1946 mutant at 45 ˚C, after incubation in 7H9 in the presence of 0.1 % SDS, pH 4.5, PBS were determined

Primer used for sequencing transposon mutants:

Mycomarseq: CTT CTG AGC GGG ACT CTG GGG

Primers for complementation constructs:

msm1946_ORF_F: GCT AGC AGC GAA CAC CGC ACG TTC GGG CTC

msm1946_ORF_R: TCA GAT ATC GTC GAG TGC GGC CCA GGA TTC GAT

Primers for qRT-PCR

SigA_F: TCG ACT ACA CCA AGG GCT AC

SigA_R: TGA TCA CCT CGA CCA TGT GC

msm1946_qPCR_F: GTG CAG TAC CTC GGC AGT CA

msm1946_qPCR_R: GCC GAT CGC GTG GAA TC
